# Influence of Donors’ and Recipients’ HLA Typing on Renal Function Immediately After Kidney Transplantation

**DOI:** 10.5812/numonthly.12328

**Published:** 2013-11-13

**Authors:** Zohreh Rostami, Nasrollah Shafighiee, Mohammad Mahdi Baghersad, Behzad Einollahi

**Affiliations:** 1 Nephrology and Urology Research Center, Baqiyatallah University of Medical Sciences, Tehran, IR Iran

**Keywords:** Histocompatibility Testing, Kidney Transplantation, Delayed Graft Function

## Abstract

**Background:**

The human leukocyte antigen (HLA) system is widely used as a strategy in the search for the etiology of renal function impairment.

**Objectives:**

This study was carried out to detect the most common HLA alleles’ distribution in kidney transplant in both donors and recipients, and clarify the association between HLA alleles and renal dysfunction immediately after transplantation.

**Patients and Methods:**

HLA-class I and II alleles typing by PCR-SSOP was performed on a total of 874 recipients aged 40.7 ± 13.8 (male/female: 562/279) and 874 donor aged 27.5 ± 5.3 (male/female: 683/110), between 2006 and 2009 in Baqiyatallah, hospital, Tehran, Iran. In this retrospective, cross sectional study, data were obtained from personal files. Donors aged 40.9 ± 13.6 years and male/female 390/195, while recipients had a mean age 27.5 ± 5.3 and male/female 523/83. Renal dysfunction defined as acute rejection, acute tubular necrosis and Delay graft function.

**Results:**

In this study common alleles at each of the loci for the human leukocyte antigen (HLA) class I (A, B, and C) and class II (DR and DQ) were A2 (n = 186, 33.8%), Bw6 (n = 196, 47.5%), Cw4 (n = 164, 39.7%), DR52 (n = 161, 29.6%), DQ3 (n = 101, 40.1%) for donors; while A2 (n = 200, 34%), BW6 (n = 235, 38.8%), Cw6 (n = 23, 15.2%), DR511 (n = 174, 30.4%), DQ1 (n = 99, 46.3%) for recipients. We detected a total of 139 case of renal dysfunction among RTRs. By the way only cold ischemic time (P = 0.03) and severe anemia (P = 0.000) were significantly associated with renal dysfunction early post kidney transplantation.

**Conclusions:**

We can predict high risk groups before kidney transplantation and try to establish a screening program for the detection of genetic susceptibility to renal function impairment. HLA typing of the donors and recipients might influence the development of new treatment strategy.

## 1. Background

Human leukocyte antigen (HLA) molecules are expressed on almost all nucleated cells (1). The HLA region on chromosome 6p21.31 comprises six major loci that express proteins which are classified into HLA class I (HLA-A, B, C) and class II (HLA-DR, -DQ, -DP) antigens. Although protection against pathogens is the primary function of HLA molecules, they are major barrier to transplantation which initiate graft rejection (2). HLA loci is one of the most polymorphic loci in the human genome, (3) but a limited number of alleles are found at a gene frequency more than 0.001 in a specified population (4). In kidney transplantation, efforts are made to match at the HLA-A, -B, and -DR loci (5).

## 2. Objectives

Due to the high polymorphism in the HLA system and since some of the HLA antigens appear to be more immunogenic, this study was designed to detect the most common distribution of HLA alleles in Iranian kidney transplant donors and recipients. The understanding of HLA subtype frequency has also permitted a better ABDR matching of donor and recipient and better clinical results. 

## 3. Patients and Methods

HLA-class I and II alleles typing by PCR-SSOP was performed on a total of 874 recipients aged 40.7 ± 13.8 (male/female: 562 /279) with median dialysis duration time 11 (0-438) months, and 874 donor aged 27.5 ± 5.3 (male/female: 683/110), between 2006 and 2009 in Baqiyatallah, hospital, Tehran, Iran. In this retrospective, cross-sectional study, allele frequencies, history of renal dysfunction, and other participants’ data were obtained from personal files by two trained medical students. Polymerase chain reaction method was used for DNA replication and HLA typing. All data were extracted from personal file.

In order to determine allele frequencies, participants’ data files were reviewed by two trained medical students. Polymerase chain reaction method was used for DNA replication and HLA typing. Two or more alleles that express the similar antigen were defined as a single allele.

### 3.1. Renal dysfunction

The definition of acute rejection was based on the conventional histological and clinical criteria of the reporting centers. Acute tubular necrosis (ATN) was defined clinically and histologically.

Delay graft function (DGF) means dialysis requirement during the first week after kidney transplantation. World Health Organization criteria, has defined a hemoglobin (Hb) level less than 11 g/dL for men and less than 10 g/dL for women as severe anemia. Body mass index (BMI) was defined in weight (kg)/height (m^2^).

### 3.2. Statistics

Data were analyzed with SPSS software (version 17.0). Quantitative variables were expressed as mean ± standard deviation, whereas qualitative variables were shown as number and percentage. Continuous data were compared by Student’s t-test, and categorized data were analyzed using the Chi-square or Fisher’s exact test.

## 4. Results

In the current study, common alleles at each loci for the human leukocyte antigen (HLA) class I (A, B, and C) and class II (DR and DQ) are summarized in Table 1. We identified 17 distinct alleles for HLA A, 33 alleles for HLA B and 7 alleles at the HLA C locus, whereas a total of 21 alleles and 13 alleles for both DR and DQ loci, respectively. We detected a total of 139 case of renal dysfunction among RTRs, with mean age of 40.9 ± 12.9 and male/female ratio of 86/48. In addition panel reactivity (more than 40%) significantly is more frequent in recipients who have DQ1 allele (P = 0.02). Mean cold and warm ischemic time were (21.3 ± 16.2) and (16.4 ± 1.7) respectively. 

**Table 1.** The Most Frequent Alleles in Donors and Recipients

HLA	Donor, No. (%)	Recipient, No. (%)
**A2**	186 (33.8)	200 (34)
**A3**	110 (20)	874 (22.1)
**Bw4**	164 (39.7)	198 (32.7)
**Bw6**	196 (47.5)	235 (38.8)
**Cw4**	79 (8.4)	9 (7)
**Cw6**	22 (2.3)	23 (15.2)
**DR11**	78 (14.4)	174 (30.4)
**DR52**	161 (29.6)	149 (25.3)
**DQ1**	95 (42.6)	99 (46.3)
**DQ3**	101 (40.1)	83 (36.2)

The most common alleles in diabetes mellitus, hypertension and autosomal dominant poly cystic kidney disease were summarized in Table 2. 

**Table 2.** The Most Common Alleles in Diabetes Mellitus, Hypertension and Autosomal Dominant Poly Cystic Kidney Disease

ESRD Etiology No. (%)	Frequency	The Most Common Alleles				
**DM**^**a**^	164 (27.8)	A2	BW4	CW4	DR11	DQ3
**HTN**^**a**^	311 (52.8)	A2	BW6	CW6	DR52	DQ1
**ADPKD**^**a**^	54 (9.2)	A2 = A3	BW4 = BW6	CW4	DR11	DQ1
**Others**	60 (10.2)					

^a^ Abbrviations: DM, diabetes mellitus; HTN, hypertension; ADPKD, autosomal dominant poly cystic kidney disease; ESRD, end stage renal disease.

In the univariate analysis, only long cold ischemic time (23.9 ± 19.7 vs. 20.6 ± 15.09, P = 0.03) and severe anemia (110 vs. 21, P = 0) were associated with renal dysfunction. ATN (12.3 ± 2.1 vs. 6.2 ± 0.2, P = 0.000) and DGF (24.7 ± 3.5 vs. 21.2 ± 1.2, P = 0.04) were frequent in long cold ischemic time. Association between ATN, DGF and acute rejection with other variables such as recipients and donors’ age, gender, BMI, donor source and warm ischemic time were not significant. We revealed demographic differences and HLA distribution among ATN, DGF and acute rejection in Table 3. Severe anemia detected in 555 (67.7%) of recipients. 

**Table 3.** Renal Dysfunction According to Demograghic and Common HLA Distibution Among Donors and Recipients.

	ATN^a^	Acute Rejection	DGF^a^
**Number**	36	96	56
**Living donor source **	30	96	47
**Donor gender**			
Male	31	83	42
Female	5	13	14
**Recipient gender**			
Male	23	66	32
Female	13	30	24
**Donor age, Mean (SD), y**	41.2 (12.6)	39 (12.7)	39.9 (15.9)
**Recipient age, Mean (SD), y**	27.3 (4.7)	27.8 (5.6)	38 (27.6)
**Warm ischemic time, Mean (SD), min**	16.4 (2.2)	16.5 (2.1)	16.9 (1.9)
**Cold ischemic time, Mean (SD), min**	23.8 (12.3)	21.2 (10.8)	30.1 (24.7)
**Severe anemia No. (%)**	29 (80.6)	75 (85.2%)	47 (88.7%)
**The most common donor HLA **			
	A2 = 9	A2 = 25	A2 = 12
	BW4 = 12	BW6 = 20	B35 = 9
	CW2 = 3	CW4 = 13	CW6 = 4
	DR11 = DR13 = 5	DR52 = 36	DR52 = 14
	DQ1 = 3	DQ1 = 23	DQ1 = 7
**The most common recipient HLA**			
	A2 = 14	A2 = 34	A2 = 16
	BW6 = 11	BW6 = 22	BW4 = 9
	CW2 = 4	CW4 = 14	CW1 = CW4 = 5
	DR4 = 9	DR52 = 29	DR53 = 18
	DQ1 = 6	DQ1 = 25	DQ1 = 12

^a^ Abbreviations: ATN, acute tubular necrosis; DGF, delay graft function

## 5. Discussion

HLA antigens expressed on the cell membrane encompass an important role in the immune response to grafted tissue. In addition, distribution of HLA allelic subtypes vary in different populations so, information about the distribution of HLA genotypes in different populations may be important because probability of incompatibility at the allele level will be affected by different origins of recipients and donors (6) and theoretically, highly compatible donor can prevent graft rejection and increase the graft survival rate (1, 7).

In transplantation, frequencies of HLA genotypes can be used to predict the chance of detecting a highly HLA match donor for an exacting recipient and to determine the effect of HLA mismatching on different renal transplant complications (8). In the unrelated renal transplantation, it is well known that high HLA matching decreases the risk of post-transplant complications, although the relative role of individual loci remains unknown (6). Since HLA system has wide-ranging polymorphism, large population samples are needed to conclude an acceptable estimation of allele frequencies. HLA typing for organ transplantation has supplied a large populations examined as donors or recipients for HLA data analysis (8). While more than 1300 alleles were defined for HLA at the DNA sequence level and several allelic subtypes were detected for most of them, a limited number of alleles have been identified in a specific population (6). As HLA A, HLA B and HLA DR have the most prominent impact on transplantation, (1, 5) this study represents the most common HLA present in both class I and class II loci in Iranian population which is illustrated in Table 1. A2, Bw6 and DR52 are the most common in donors whereas A2, Bw6 and DR11 are the most common in recipients. By the way, we concluded several complications were more frequent in recipients with HLA A3, Bw4 and Bw6. But we have to confirm these finding by large prospective studies too. 

Conclusion: HLA typing of the donors and recipient might influence the development of new treatment strategy; additionally it provides useful data for HLA matching in transplantation. We can predict high risk group before kidney transplantation and try to establish a screening program for the detection of genetic susceptibility to renal function impairment. HLA typing of the donors and recipient might influence the development of new treatment strategy.
